# Region-Wise Distribution of Schizophrenia With Cannabis Abuse and Medication Non-Compliance in the United States: A Nationwide Analysis of 51,975 Hospitalizations

**DOI:** 10.7759/cureus.7936

**Published:** 2020-05-02

**Authors:** Ramu Vadukapuram, Shaheer Zahid, Hyun Kyung Lee, Rikinkumar S Patel

**Affiliations:** 1 Psychiatry, State University of New York Upstate Medical University, Syracuse, USA; 2 Psychiatry, Saint James School of Medicine, Park Ridge, USA; 3 Psychiatry, Hanyang University College of Medicine, Seoul, KOR; 4 Psychiatry, Griffin Memorial Hospital, Norman, USA

**Keywords:** schizophrenia, medication noncompliance, cannabis use, marijuana use, hospital epidemiology, substance abuse, medication non-adherence, psychotic disorder

## Abstract

Objectives

To understand the region-wise differences in demographics, comorbid substance abuse, and hospital outcomes in adult schizophrenia inpatients with cannabis abuse and medication non-compliance.

Methods

We included 51,975 adults (18-65 years) from the Nationwide Inpatient Sample (2012 to 2014) with a primary diagnosis of schizophrenia and comorbid diagnosis of cannabis abuse and medication non-compliance. We used descriptive statistics and linear-by-linear association to evaluate the region-wise differences in demographics and comorbid substance abuse. Analysis of variance was used for continuous variables such as length of stay (LOS) and total charges during hospitalization to measure the differences across the regions.

Results

Our study inpatients were from the United States regions: northeast ([NE] 30.4%), midwest ([MW] 24.3%), south (27.3%), and west (18%). A higher proportion of young adults (age: 18-35 years; overall total: 62.4%) were from the south (65.1%) and the NE (64.3%) regions. The study population comprised majorly of males in all the regions, ranging from 78.6% to 82.2% (overall total: 80.5%). The west region comprised majorly of whites (42.6%), whereas all other regions majorly had blacks, with the highest seen in the MW (63.2%) and south (63%) regions. The most prevalent comorbid substances in the study inpatients were tobacco (46.3%) and alcohol (32.3%). The mean LOS and total charges for the hospitalization were much higher in the NE region (LOS: 15.8 days; total charges: $44,336).

Conclusion

Cannabis abuse and medication non-compliance in schizophrenia patients were prevalent in the NE region of the United States and in the overall regions, and affects young adults, males, and Blacks from low-income families. This is associated with higher hospitalization stay and cost, which indirectly increase the healthcare burden.

## Introduction

Schizophrenia is a complex psychiatric illness characterized by a disruption in the thought process, perception, emotional responsiveness, and social interaction. The age of onset is usually mid-twenties in men and in the late twenties in women [1]. The prevalence of schizophrenia is about 1% globally and is 1 of the top 15 leading causes of disability [2,3]. Schizophrenia has a huge impact on healthcare and is an economic burden; as reported in 2013, it was $155.7 billion including 24% of direct medical service cost, 6% of direct non-medical service cost, and 76% of indirect medical service cost [4].

Second-generation antipsychotics (SGAs) were introduced in 1990 in the United States (US); since then, SGAs are used as first-line agents for treating schizophrenia due to increased efficacy for both positive and negative symptoms, and safety profile [5]. Medication non-compliance is a major concern in schizophrenia management as it leads to significant suffering to the patients as well as their families, with a rising healthcare burden [6]. Of the 1.2 million schizophrenia patients registered in the Medical Expenditure Panel Survey from 2010 to 2014, more than 70% of the patients were non-adherent to antipsychotics [6]. In a systemic review of nine research studies including 2,643 participants, the prevalence of medication non-compliance among schizophrenia patients was around 56% [7]. Antipsychotic medication non-compliance can result in increased hospitalization stay and higher healthcare costs [8,9]. The national re-hospitalization cost in the U.S. due to antipsychotic non-compliance was about $1,500 million [8-10].

Substance use disorder (SUD) is a major concern in schizophrenia patients, with cannabis being the most preferred substance of choice [11]. As per a longitudinal study, heavy cannabis use can be a major risk factor by six times for schizophrenia [12]. In a meta-analysis of 123 studies with 165,811 schizophrenia patients, the prevalence of any SUD and cannabis abuse was 41.7% and 26.2%, respectively [13]. The relationship between higher cannabis use and schizophrenia is still not clear. Cannabis use can lead to schizophrenia, as seen in a 15-year follow-up study of 45,570 participants, as patients who consumed cannabis more than 50 times had a six times higher risk of schizophrenia [12]. In a review of 15 observational studies (N = 3,678), they found an increased medication non-compliance in cannabis users by 2.5 times compared with the non-users [14]. They also found an increased medication non-compliance in current cannabis users by 5.8 times compared with non-users and by 5.5 times compared with former cannabis users [14].

In our study, we used the national inpatient data from the U.S. hospitals to understand the region-wise differences in demographics, comorbid substance abuse, and outcomes (hospitalization stay and charges) in adult schizophrenia inpatients with cannabis abuse and medication non-compliance.

## Materials and methods

Data source

**Figure 1:**
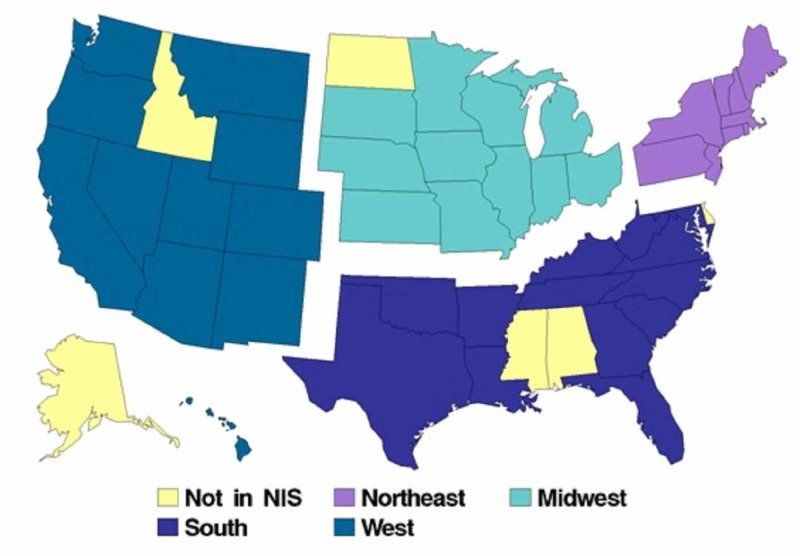
States by region Source: region-wise division per the U.S. Census Bureau [16].

We conducted a retrospective study using the Nationwide Inpatient Sample (NIS) data from January 2012 to December 2014 from the Healthcare Cost and Utilization Project (HCUP) [15]. The NIS provides patient health records from around 4,400 non-federal and community-based hospitals across 44 states in the U.S. Census region was obtained using the American hospital association annual survey of hospitals [15]. Diagnostic information in the NIS is identified using the International Classification of Diseases, Ninth Edition (ICD-9) codes and the Clinical Classification Software (CCS) codes [15]. As per the U.S. Census Bureau, the states included in each region are as follows: northeast (NE), midwest (MW), south, and west (Figure 1) [16].

Inclusion criteria and outcome variables

We included adult inpatients (age: 18-65 years) with a primary discharge diagnosis of schizophrenia and other psychotic disorders using the CCS code 659. This was further focused by including only inpatients with comorbid cannabis abuse (ICD-9 codes: 304.30, 304.31, 304.32, or 305.2) and a history of medication non-compliance (ICD-9 code: V15.81).

The demographic characteristics included age (18-35, 36-50, and 51-65 years), sex (male or female), race (white, black, Hispanic, and Asian/native American), and median household income (based on percentiles) [16]. The comorbid substance included alcohol abuse, tobacco abuse, amphetamine abuse, cocaine abuse, and opioid abuse, which were identified using ICD-9 diagnosis codes [16]. Length of stay (LOS) in the NIS is calculated as a number of nights the patient stayed in the hospital for the management of schizophrenia, and total charges do not include professional fees and/or other non-covered charges during hospitalization [16].

Statistical analysis

We used descriptive statistics and linear-by-linear association to evaluate the region-wise differences in demographic and comorbid substance abuse in the study inpatient population. Analysis of variance was used for the continuous variables (age, LOS, and total charges) to measure the differences across the regions. A P-value of less than 0.01 was used to decide the statistical significance in the data analyses conducted using SPSS Version 26 (IBM Corp., Armonk, NY, USA).

Ethical approval

Individual identifiers were used to protect patient health information, and therefore this is a de-identified database that does not require the Institutional Review Board approval [15].

## Results

We analyzed data of 51,975 schizophrenia inpatients with cannabis abuse and medication non-compliance (i.e. our study population) from the following U.S. regions: NE (30.4%), MW (24.3%), south (27.3%), and west (18%). A higher proportion of young adults (age: 18-35 years; overall total: 62.4%) were from the south (65.1%) and the NE (64.3%) regions, whereas older-age adults (51-65 years) were from the south (12.9%) and the MW (12.8%) regions. The study population comprised majorly of males in all the regions ranging from 78.6% to 82.2% (overall total: 80.5%). The west region comprised majorly of whites (42.6%), whereas all other regions majorly had blacks, with the highest seen in the MW (63.2%) and south (63%) regions. Hispanics comprised 10.1% of total inpatients but were seen in about one-fifth of the west region (21.5%). The overall study population was from low-income families below the 25th percentile (48.6%), but it was seen much higher in the MW (59.2%) and south (51.3%) regions. And, the prevalence was lowest (12.7%) in high-income families above 75th percentile, though it was much higher than the overall total in the NE (18%) and west (16.6%) regions. The demographic differences across the regions were statistically significant (P < 0.001). The study population was majorly covered by public insurances: Medicaid (48.1%) and Medicare (28%) (Table 1).

**Table 1:** Region-wise distribution of schizophrenia inpatients with cannabis abuse and medication non-compliance

Variable	Northeast	Midwest	South	West	Total	P-value
Total, N	15,805	12,610	14,170	9,390	51,975	-
Prevalence, %	30.4	24.3	27.3	18	-	-
Mean age, years	33.4	35.1	34.3	34.3	34.2	<0.001
Age at admission, %						
18–35 years	64.3	58.1	65.1	60.9	62.4	<0.001
36–50 years	26.4	29.1	22.0	26.5	25.9	
51–65 years	9.4	12.8	12.9	12.6	11.7	
Sex, %						
Female	17.8	21.2	18.8	21.4	19.5	<0.001
Male	82.2	78.8	81.2	78.6	80.5	
Race, %						
White	24.6	28.7	28.1	42.6	29.8	<0.001
Black	54.5	63.2	63.0	28.9	54.1	
Hispanic	12.2	2.8	5.8	21.5	10.1	
Native American/Asian	8.7	5.3	3.0	6.9	6.1	
Median household income in percentiles, %						
0–25th	45.9	59.2	51.3	31.6	48.6	<0.001
26th–50th	19.7	20.9	22.3	28.1	22.2	
51st–75th	16.4	13.4	15.3	23.7	16.6	
76th–100th	18.0	6.4	11.0	16.6	12.7	
Primary payer, %						
Medicare	24.5	33.9	29.0	24.3	28.0	<0.001
Medicaid	54.6	44.4	41.6	51.7	48.1	
Private	11.4	10.5	12.9	9.4	11.2	
Uninsured/self-pay	9.4	11.2	16.4	14.7	12.7	

**Figure 2:**
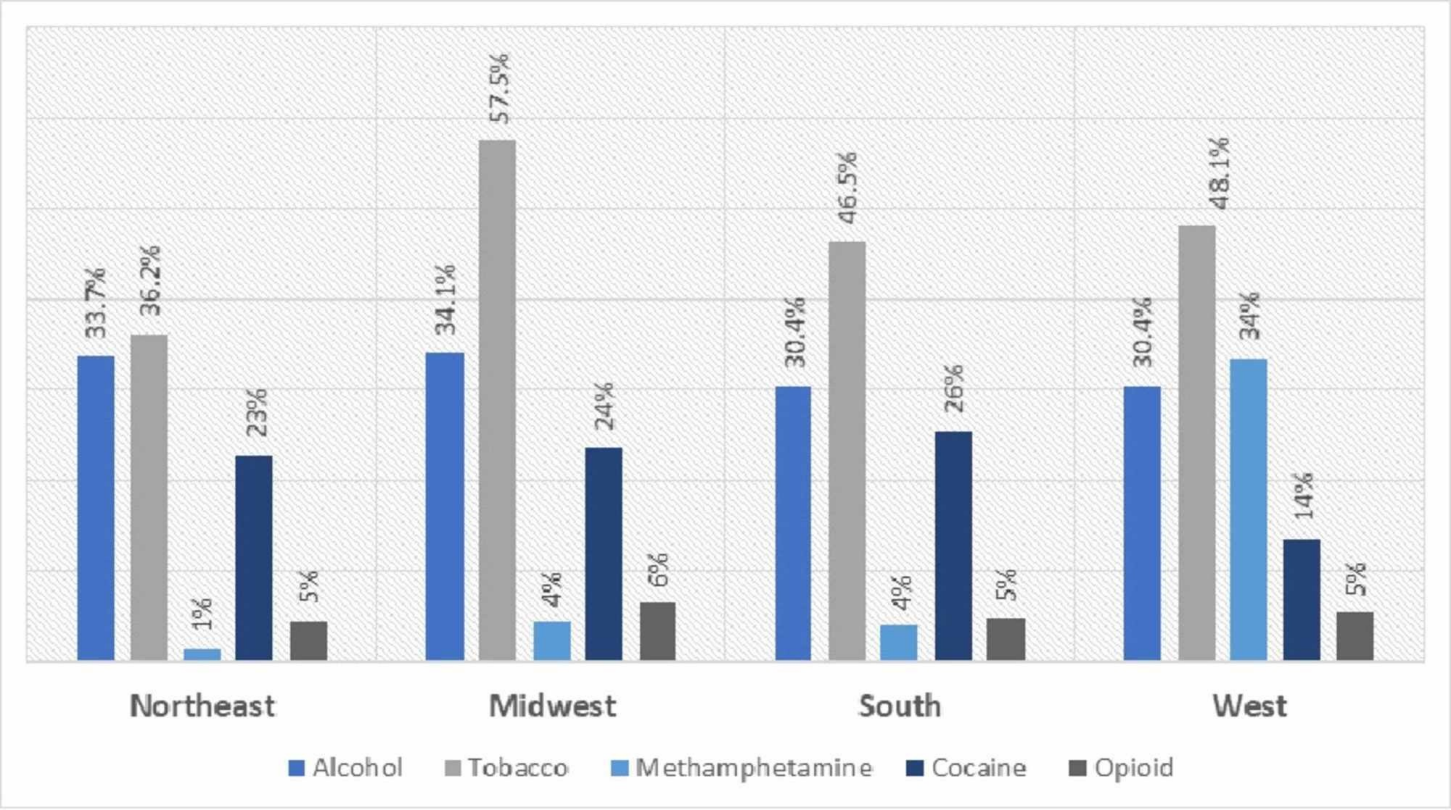
Region-wise distribution of comorbid substance abuse

The most prevalent comorbid substances in the study population were tobacco (46.3%) and alcohol (32.3%), with a higher rate seen in the MW region (tobacco abuse: 57.5%; alcohol abuse: 34.1%). Opioid abuse was the less preferred substance in the study population (4.5% in NE and 6.4% in MW) and there was statistically no significant difference across the regions (P = 0.051). Among the stimulants, cocaine abuse was more prevalent (22%) versus methamphetamine abuse (8.6%) in the overall population, but the trend was different in the west region, with methamphetamine abuse (33.5%) being greater than cocaine abuse (13.5%) (Figure 2).

**Figure 3:**
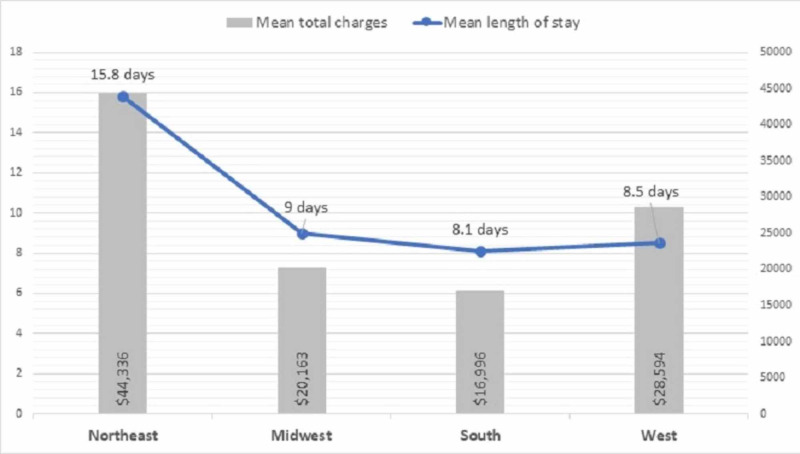
Region-wise impact on hospital length of stay and total charges

The mean LOS and total charges for the hospitalization were much higher in the NE region (LOS: 15.8 days; total charges: $44,335.9), whereas it was lowest in the south region (LOS: 8.1 days; total charges: $16,996.2) (Figure 3).

## Discussion

In our study population of schizophrenia inpatients with cannabis abuse and medication non-compliance, around 30.4% inpatients were from the NE region of the U.S., with the lowest in the west region (18%). A similar geographical distribution was seen in a meta-analysis (N = 420,000; age: 12-24 years) by Leinweber et al. with an estimated annual incidence of cannabis use being highest in the NE region (9%) and the lowest in the west region (3%) [17]. Also, the medication non-compliance rate was high in the south region and lowest in the MW region (40.24% vs. 15.35%; P = 0.003) [6].

About three-fifths of our study inpatients were young adults (18 to 35 years), with the majority of the patients from the south (65.1%) and NE (64.3%) regions in this age group. Also, self-reported cannabis use was seen more in younger individuals (19-20 years) with the risk of developing cannabis use disorder (CUD) over a period of 15 years [18]. Young adults had four “typical” reasons for cannabis use, including “to feel good/get high”, “to have a good time with friends”, “to experiment”, and “to relax” [18]. Overall, 80.5% (range: 78.6-82.2% per region) cannabis user males had medication non-compliance issue. A nearly similar result was seen in a cross-sectional survey of cannabis users (N = 2,374), with 57.7% men with medication non-compliance, though there existed a significant difference in cannabis addictiveness, with a much higher percentage in females (17.5% vs. 11.3% in males; P <0.001) [19]. This probably could be due to the reason that females may lack knowledge about the addictive nature of cannabis [19]. Males with schizophrenia were more non-compliant with antipsychotic medications compared with females (51.84% vs. 48.15%; P = 0.01) [6]. But, a study by Acosta et al. found a non-significant association between gender and medication non-compliance (77.8% in men vs. 22.2% in women; P = 0.351) [20].

Among races, a higher proportion (54.1%) of blacks had medication non-compliance and cannabis use, with a much lower prevalence in the whites. This was contrary to the past study that found that the whites were more non-compliant to regimen compared with non-whites [6]. Also, patients from poor socioeconomic status had a high prevalence (69.9%) of medication non-compliance compared with those from high socioeconomic status (11.2%) [6]. These findings correlate with our study findings of 48.6% in patients from low-income families below 25th percentile versus 12.7% in those from median income above 75th percentile income. Also, a longitudinal cohort study including 1,037 individuals followed from birth to ages 18 to 38 years found that those with regular cannabis use faced drifting of socioeconomic status toward the lower side along with workplace and relationship problems in early midlife [21].

The most prevalent comorbid substances in our study inpatients were tobacco (46.3%) and alcohol (32.3%). This finding was consistent with another national study (N = 6,624) that found that 44.7% of lifetime cannabis users can use any other illicit substance, which may be related to the severity of the current substance use and comorbid psychiatric disorders [22]. A nationwide survey of self-reported adult schizophrenia patients (N = 876) found that medication adverse effects such as extrapyramidal symptoms, tardive dyskinesia, agitation, and sedation were significantly associated medication non-compliance [23,24].

A study of 9,889 schizophrenia patients found that young adults (18 to 35 years) had higher inpatient costs ($15,692 vs. $10,274; P < 0.001) and longer LOS (9.6 vs. 5.9 days; P < 0.001) compared with older adults [25]. In our study, the longer hospitalization stay and the higher cost were seen in the NE region, with mean LOS of 16 days and total charges of $44,336 per hospitalization. This possibly could be because around three-fifths of the population in the NE region comprise young adults. Also, young adults have more psychiatric comorbidities compared with older individuals with schizophrenia, which may impact psychiatric care and hospital LOS [25].

Our study has few limitations such as NIS being an administrative database deficient of the patient-level information with a higher possibility of selection bias. Due to the cross-sectional nature of the study, we were not able to address the impact of medication non-compliance during and after receiving psychiatric inpatient care. We were not able to interpret re-hospitalizations, which may add to the total inpatient visits in the study. Despite these limitations, using the NIS we provided an exclusive population-based perspective on the trend of region-wise hospitalization of adult schizophrenia inpatients with cannabis abuse and medication and non-compliance.

## Conclusions

Cannabis abuse and medication non-compliance in schizophrenia patients were prevalent in the NE region of the U.S. and in the overall regions, and affects young adults, males, and blacks from low-income families. This is associated with higher hospitalization stay and cost due to which both CUD and medication non-compliance indirectly increase the healthcare burden and worsen the quality of life in schizophrenia patients.

## References

[1] (2013). Diagnostic and Statistical Manual of Mental Disorders, Fifth Edition.

[2] Moreno-Küstner B, Martín C, Pastor L (2020). Prevalence of psychotic disorders and its association with methodological issues. a systematic review and meta-analyses. PLoS One.

[3] GBD 2016 Disease and Injury Incidence and Prevalence Collaborators (2017). Prevalence, global, regional, and national incidence, prevalence, and years lived with disability for 328 diseases and injuries for 195 countries, 1990-2016: a systematic analysis for the global burden of disease study 2016. Lancet.

[4] Cloutier M, Aigbogun MS, Guerin A (2016). The economic burden of schizophrenia in the United States in 2013. J Clin Psychiatry.

[5] Shen WW (1999). A history of antipsychotic drug development. Compr Psychiatry.

[6] Desai R, Nayak R (2019). Effects of medication nonadherence and comorbidity on health resource utilization in schizophrenia. J Manag Care Spec Pharm.

[7] Semahegn A, Torpey K, Manu A, Assefa N, Tesfaye G, Ankomah A (2020). Psychotropic medication non-adherence and its associated factors among patients with major psychiatric disorders: a systematic review and meta-analysis. Syst Rev.

[8] Sun SX, Liu GG, Christensen DB, Fu AZ (2007). Review and analysis of hospitalization costs associated with antipsychotic nonadherence in the treatment of schizophrenia in the United States. Curr Med Res Opin.

[9] Dilla T, Ciudad A, Alvarez M (2020). Systematic review of the economic aspects of nonadherence to antipsychotic medication in patients with schizophrenia. Patient Prefer Adherence.

[10] Haddad PM, Brain C, Scott J (2020). Nonadherence with antipsychotic medication in schizophrenia: challenges and management strategies. Patient Relat Outcome Meas.

[11] Rodrigo C, Rajapakse S (2009). Cannabis and schizophrenia spectrum disorders: a review of clinical studies. Indian J Psychol Med.

[12] Andréasson S, Allebeck P, Engström A, Rydberg U (1987). Cannabis and schizophrenia. a longitudinal study of Swedish conscripts. Lancet.

[13] Hunt GE, Large MM, Cleary M, Lai HMX, Saunders JB (2018). Prevalence of comorbid substance use in schizophrenia spectrum disorders in community and clinical settings, 1990-2017: systematic review and meta-analysis. Drug Alcohol Depend.

[14] Foglia E, Schoeler T, Klamerus E, Morgan K, Bhattacharyya S (2017). Cannabis use and adherence to antipsychotic medication: a systematic review and meta-analysis. Psychol Med.

[15] (2020). Overview of the national (nationwide) inpatient sample (NIS). https://www.hcup-us.ahrq.gov/nisoverview.jsp.

[16] (2020). NIS description of data elements. https://www.hcup-us.ahrq.gov/db/nation/nis/nisdde.jsp.

[17] Leinweber JP, Cheng HG, Lopez-Quintero C, Anthony JC (2020). Newly incident cannabis use in the United States, 2002-2011: a regional and state level benchmark. Peer J.

[18] Patrick ME, Bray BC, Berglund PA (2016). Reasons for marijuana use among young adults and long-term associations with marijuana use and problems. J Stud Alcohol Drugs.

[19] Cuttler C, Mischley LK, Sexton M (2020). Sex differences in cannabis use and effects: a cross-sectional survey of cannabis users. Cannabis Cannabinoid Res.

[20] Acosta FJ, Bosch E, Sarmiento G, Juanes N, Caballero-Hidalgo A, Mayans T (2009). Evaluation of noncompliance in schizophrenia patients using electronic monitoring (MEMS) and its relationship to sociodemographic, clinical and psychopathological variables. Schizophr Res.

[21] Cerdá M, Moffitt TE, Meier MH (2016). Persistent cannabis dependence and alcohol dependence represent risks for midlife economic and social problems: a longitudinal cohort study. Clin Psychol Sci.

[22] Secades-Villa R, Garcia-Rodríguez O, Jin CJ, Wang S, Blanco C (2015). Probability and predictors of the cannabis gateway effect: a national study. Int J Drug Policy.

[23] Dibonaventura M, Gabriel S, Dupclay L, Gupta S, Kim E (2020). A patient perspective of the impact of medication side effects on adherence: results of a cross-sectional nationwide survey of patients with schizophrenia. BMC Psychiatry.

[24] Patel RS, Mansuri Z, Chopra A (2020). Analysis of risk factors and outcomes in psychiatric inpatients with tardive dyskinesia: a nationwide case-control study. Heliyon.

[25] Huang A, Amos TB, Joshi K, Wang L, Nash A (2018). Understanding healthcare burden and treatment patterns among young adults with schizophrenia. J Med Econ.

